# Preclinical Pharmacokinetics, Tissue Distribution, and Primary Safety Evaluation of Indo5, a Novel Selective Inhibitor of c-Met and Trks

**DOI:** 10.3389/fphar.2021.711126

**Published:** 2021-08-10

**Authors:** Teng Luo, Fei-Xiang Zhang, Ke Zhao, Hui-Ying Gao, Shou-Guo Zhang, Lin Wang, Gui-Fang Dou, Ting Liu, Miao Yu, Yi-Qun Zhan, Hui Chen, Xiao-Ming Yang, Chang-Yan Li

**Affiliations:** ^1^ State Key Laboratory of Proteomics, Beijing Proteome Research Center, National Center for Protein Sciences (Beijing), Beijing Institute of Lifeomics, Beijing, China; ^2^ Beijing Institute of Radiation Medicine, Beijing, China; ^3^ School of Basic Medical Sciences, Anhui Medical University, Hefei, China; ^4^ Department of Pharmaceutical Engineering, School of Chemical Engineering and Technology, Tianjin University, Tianjin, China; ^5^ Institute of NBC Defence, Beijing, China

**Keywords:** Indo5, hepatocellular carcinoma, tissue distribution, toxicity, pharmacokinetic study

## Abstract

The compound [3-(1H-benzimidazol-2-methylene)-5-(2-methylphenylaminosulfo)-2-indolone], known as Indo5, is a novel selective inhibitor of c-Met and Trks, and it is a promising anticancer candidate against hepatocellular carcinoma (HCC). Assessing the pharmacokinetic properties, tissue distribution, and toxicity of Indo5 is critical for its medicinal evaluation. A series of sensitive and specific liquid chromatography-tandem mass spectrometry methods were developed and validated to determine the concentration of Indo5 in rat plasma and tissue homogenates. These methods were then applied to investigate the pharmacokinetics and tissue distribution of Indo5 in rats. After intravenous injection of Indo5, the maximum concentration (C_max_) and the time at which C_max_ was reached (T_max_) were 1,565.3 ± 286.2 ng/ml and 1 min, respectively. After oral administration, C_max_ and T_max_ were 54.7 ± 10.4 ng/ml and 2.0 ± 0.48 h, respectively. We calculated the absolute oral bioavailability of Indo5 in rats to be 1.59%. Following intravenous injection, the concentrations of Indo5 in various tissues showed the following order: liver > kidney ≈ heart > lung ≈ large intestine ≈ small intestine ≈ stomach > spleen > brain ≈ testes; hence, Indo5 distributed highest in the liver and could not cross the blood–brain or blood–testes barriers. Continuous injection of Indo5 for 21 days did not lead to liver injury, considering unchanged ALT and AST levels, normal histological architecture of the liver, and normal number and frequencies of immune cells in the liver, indicating a very low toxicity of Indo5 *in vivo*. Collectively, our findings provide a comprehensive understanding of the biological actions of Indo5 *in vivo and further support* its development as an antitumor treatment for HCC patients.

## Introduction

Hepatocellular carcinoma (HCC) accounts for about 80–90% of primary liver cancers (Llovet et al., 2008; Pan et al., 2011; Stewart and Wild, 2014; Sia et al., 2017). The incidence and mortality rates of HCC have been increasing for decades, with approximately 800,000 new cases occurring every year worldwide (Sia et al., 2017; Bertot and Adams, 2019). Limited treatment options with marginal clinical benefits are available for patients with HCC. Systemic therapy, particularly in the form of conventional cytotoxic drugs, is, in general, ineffective. In recent years, molecular-targeted therapies have been used to treat various types of cancer, including liver cancer. This approach inhibits the growth of tumor cells by interfering with molecules involved in carcinogenesis; thus, molecular-targeted therapy is more selective and specific than cytotoxic chemotherapy (Alqahtani et al., 2019). The first targeted, systemic therapy approved for the treatment of advanced HCC by the US Food and Drug Administration (FDA) in 2007 was based on the multi-tyrosine kinase inhibitor sorafenib (Shen et al., 2013; Stotz et al., 2015). Nevertheless, sorafenib continued to show severe treatment-related adverse effects, such as diarrhea, nausea, vomiting, anorexia, fatigue, hand-and-foot skin reactions, hypertension, bleeding risk, and kidney toxicity (Llovet et al., 2008; Cheng et al., 2009; Li et al., 2015a). Regorafenib is the second-line targeted therapy for HCC and shows several adverse effects, such as severe liver injury, hemorrhage, gastrointestinal perforation, dermatological toxicity, and cardiac ischemia (George et al., 2012; Bruix et al., 2013; Grothey et al., 2013; Bruix et al., 2017). The inability of HCC patients to tolerate adverse effects often leads to a reduction in the doses of these drugs or cessation of their use, thereby diminishing the treatment efficacy. Therefore, development of novel molecular-targeted therapies for HCC with low toxicity or fewer adverse effects is urgently required.

The compound (3-(1H-benzimidazol-2-methylene)-5-(2-methylphenylaminosulfo)-2-indolone), known as Indo5, is a novel lead compound containing an indolone core, which has been identified as a versatile scaffold for the development of protein kinase inhibitors (Sun et al., 1998). Indo5 has been shown to selectively inhibit the kinase activities of c-Met, TrkA, and TrkB with a half-maximal inhibitory concentration of 14.37, 28, and 25 nM, respectively, (Luo et al., 2019). Indo5 has been shown to abrogate HGF-induced c-Met signaling activation and BDNF/NGF-induced Trks signaling activation, as well as c-Met or TrkB-mediated cell transformation and migration. Furthermore, Indo5 significantly decreases the growth of HCC cells in xenograft-transplanted mice and increases the survival of mice with hepatic orthotopic tumors. Those findings have indicated that Indo5 is associated with marked suppression of HCC cells co-expressing c-Met and Trks, which supports the clinical development of Indo5 as an antitumor treatment for HCC patients with co-active c-Met and Trks signaling. Hence, a thorough understanding of the biological actions of Indo5 *in vivo* is critical for its medical application.

Here, we investigate the preclinical pharmacokinetics, tissue distribution, and primary safety of Indo5 *in vivo*. Our findings further support its development as an antitumor treatment for HCC patients.

## Materials and Methods

### Chemicals and Reagents

Indo5 was synthesized as described previously (US Patent Number: 9642839B2). Its purity was determined using high-performance liquid chromatography (HPLC), and the compound was used as the reference standard because of its high purity (>99%). The molecular structure of Indo5 was characterized using Fourier-transform infrared (FT-IR) spectroscopy, ^1^H and ^13^C nuclear magnetic resonance (^1^H and ^13^C NMR), mass spectrometry (MS), and elemental analyses. These analyses were conducted at a laboratory specializing in pharmaceutical analysis.

The internal standard (IS), terfenadine, was purchased from Sigma-Aldrich (St. Louis, MO, United States). HPLC-grade water was obtained from Watson’s Food and Beverage (Guangzhou, China). HPLC-grade acetonitrile and HPLC-grade methanol were purchased from Thermo Fisher Scientific (Waltham, MA, United States). HPLC-grade formic acid and dimethyl sulfoxide (DMSO) were obtained from Sigma-Aldrich. The anesthetic phenobarbital for veterinary use was acquired from Wuhan Dongkang Source Technology (Wuhan, China). Heparin (1000 UI/ml) and physiologic saline (0.9%) were purchased from Sinopharm Chemical Reagents (Shanghai, China).

### Indo5 Formulation

Indo5 (10 mg) was dissolved in 10 ml DMSO. The formulation was a clear-yellow solution, which was vortexed for 3 min and sterilized by passing through a 0.22-μm filter. A final concentration of 1 mg/ml was obtained. The solution was stored at 4°C and used to manufacture standards.

### Animals

Male Wistar rats (300 ± 20 g) and mice (5–6 weeks old) were purchased from Beijing Vital River Laboratory Animal Technology (Beijing, China) and allowed to acclimate to their surroundings for 7 days. All of the animals were housed in individually ventilated cages in specific pathogen-free conditions at the animal facility of our institute. They were exposed to a 12-h light–dark cycle and allowed free access to food and water. The protocol for animal experiments was approved by the Animal Ethics Committee of the Academy of Military Medical Science (Beijing, China), and all of the experiments were conducted in accordance with the National Institutes of Health guidelines.

### Instrumentation and Analytical Conditions

#### HPLC Conditions

Chromatography was undertaken on an LC-20AD series HPLC system (Shimadzu, Kyoto, Japan) equipped with a vacuum degasser, a binary pump, an autosampler, and a thermostat column oven. Separation of Indo5 was carried out on a Biobasic-18 column (5 μm, 2.1 mm × 50 mm; Thermo Fisher Scientific) at a flow rate of 500 μl/min and column temperature of 30°C. The injection volume was 10 μl.

#### MS/MS Conditions

An API4000 quadrupole mass spectrometer (Sciex, Framingham, MA, United States) was used for the analyses. The MS/MS conditions were as follows: gas temperature, 325°C; drying gas flow, 7 L/min; nebulizer pressure, 45 psi; Vcap voltage, 3,500 V; sheath-gas temperature, 350°C; sheath-gas flow, 9 L/min; and nozzle voltage, 0 V. The transition for multiple reaction monitoring (MRM) was *m/z* 431.1→232.2 for Indo5.

Data on drug concentration in blood were collected, processed, and analyzed using Analyst 1.6 (Sciex). A linear weighting factor = 1/X^2^ model was used to process the data. An individual drug concentration in blood lower than the lower limit of quantification (LLOQ) was marked as “BQL” and recorded as zero. The above-mentioned calculations were completed using Excel™ 2016 (Microsoft, Redmond, WA, United States).

### Preparation of Calibration Standards and Quality Control Samples

Calibration standards were prepared daily by diluting stock solutions. Briefly, appropriate volumes of stock solution were spiked into 100 μl of blank plasma and tissue homogenate. Then, the samples were vortexed for 3 min. Next, appropriate volumes of acetonitrile were added until each sample reached a final volume of 500 μl; these samples were centrifuged at 10,000 g using an SL eight Small Benchtop Centrifuge (Thermo Fisher Scientific) for 10 min at room temperature. Finally, the supernatant (40 μl) was injected into the HPLC/MS system. Standard solutions were prepared in triplicate for seven calibration points, and the final concentrations obtained were 1, 2, 5, 20, 50, 100, and 500 ng/ml.

Calibration curves were constructed in triplicate by plotting known concentrations of the standard *versus* the detector response area. In the same way, a separate set of stock solutions was prepared for QC samples to determine accuracy and precision.

### Method Validation

The developed method was validated in accordance with the bioanalytical guidance from the FDA (FDA, Guidance for Industry Bioanalytical Method Validation, 2018), with determination of the following parameters: linearity; within-run and between-run accuracy and precision; recovery; LLOQ; limit of detection (LOD); selectivity; and stability.

### Pharmacokinetic Studies

For intravenous (IV) treatment, Indo5 was dissolved in DMSO (solution, 1 mg/ml) and injected into the lateral tail vein of rats (1 mg/kg, *n* = 3). For intragastric treatment, Indo5 was dissolved in 5% CMC-Na (suspension, 20 mg/ml). The rats were orally administered with Indo5 (100 mg/kg, *n* = 3) using a 20-G gavage needle. All of the animals were given a standard diet 4 h after the dosing. Blood samples (approximately 400 μl) were collected from each rat and placed into heparinized tubes before the dosing, and at 5, 15, 30, 60, 120, 240, and 480 min after the dosing. Plasma (100 μl) was harvested by centrifuging blood samples at 3,600 g for 10 min at room temperature followed by immediate processing for the analyses.

#### Sample Preparation

Briefly, 400 μl acetonitrile was added to 100 μl rat plasma or tissue homogenate, vortexed for 30 s, and centrifuged at 10,000 g for 10 min at room temperature to precipitate proteins. Subsequently, the supernatant (40 μl) was introduced into the HPLC/MS system. Plasma or tissue concentrations of Indo5 were determined using HPLC-MS/MS validated for pharmacokinetics.

#### Analyses of Pharmacokinetic Data

The plasma concentration–time data obtained after intravenous and intragastric administrations were subjected to non-compartmental analyses based on the statistical moment theory. Pharmacokinetic parameters were calculated using WinNorlin 5.2 (Certara; www.certara.com/). These parameters included the maximum plasma concentration (C_max_), time to reach C_max_ (T_max_), elimination half-life (t_1/2_), area under the plasma concentration–time curve from time zero to infinity (AUC_0–∞_), mean residence time (MRT), and clearance (CL). The absolute oral bioavailability was calculated by dividing the AUC_tot_ obtained from oral administration by the AUC_tot_ obtained from IV administration, and the calculation was adjusted using the doses that had been administered *via* oral and IV routes.

### Tissue-Distribution Study

Twelve male Wistar rats were given a single dose of Indo5 (1 mg/kg, IV). At 2, 5, 30, 60, 120, 480, and 720 min after the dosing, a group of animals (*n* = 4 for each treatment time) was sacrificed. The liver, kidneys, heart, lungs, stomach, small intestine, small-intestine content, large intestine, large-intestine content, spleen, brain, and testes were dissected rapidly and harvested. All of the tissues were rinsed thoroughly in ice-cold physiologic saline to eliminate blood and other content. The tissues and contents were processed by homogenization with 0.9% saline in a 1:3 (w/v) ratio. The preparation process for analyses was the same as that described for plasma.

### Isolation of Mononuclear Cells From Mouse Liver

Freshly separated liver was rinsed in ice-cooled phosphate-buffered saline (PBS) and then placed on a 40-μm filter. We added an appropriate amount of RPMI-1640 medium containing 2% fetal bovine serum (FBS) and used the piston of a 5-ml syringe to squeeze the tissue blocks to the bottom of the screen until only fibrous tissue remained. The volume of cell suspension collected was 40 ml. We centrifuged the cell suspension at 50 g for 5 min at 4°C and collected the supernatant. Next, we centrifuged at 800 g for 10 min at 4°C and discarded the supernatant. The precipitate was resuspended with a solution containing 40% Percoll™ (2 ml of 90% Percoll with 2.5 ml RPMI-1640 medium). We carefully added the suspension, using a capillary pipette, to a solution containing 70% Percoll (4 ml of 90% Percoll with 1.12 ml RPMI-1640 medium). Next, we centrifuged at 800 g for 20 min at 4°C, removed the upper layer, took the middle layer, and added an appropriate amount of 2% FBS in RPMI-1640 medium to reach 15 ml. Then, we undertook centrifugation at 800 g for 10 min at 4°C, removed the supernatant, and transferred it to a fresh 1.5-ml Eppendorf™ tube. Next, it was centrifuged at 1800 g for 5 min at room temperature and dried with absorbent paper. We added 300 μl of red blood cell (RBC) lysate for resuspension, lysed the RBCs for 3 min at room temperature, added 1 ml of RPMI-1640 medium with 2% FBS to stop the lysis, and centrifuged at 1800 g for 5 min at room temperature. An appropriate amount of PBS containing 2% FBS was added to resuspend the cells.

### Flow Cytometry

The cell suspension was added to a 1.5-ml Eppendorf tube, incubated with antibody for 30 min at 4°C in the dark, washed twice with 2% PBS, resuspended with 400 μl of 2% PBS, and analyzed by flow cytometry. The antibodies used for labeling were as follows: cluster of differentiation (CD)3-PerCP-Cy5.5, NK1.1-FITC, B220-APC, CD11c-FITC, CD11b-APC, CD4-APC, CD8-PE, and CD69-PE/Cy7; they were obtained from eBioscience (San Diego, CA, United States) or BD Biosciences (Franklin Lakes, NJ, United States).

### Histopathology

The tissues were fixed in 10% formalin and embedded in paraffin. Then, 4-μm-thick sections were stained with hematoxylin and eosin for histopathological and morphological analyses.

### Statistical Analysis

All of the data were expressed as mean ± standard deviation (SD) of at least three independent experiments. For comparisons between two groups, two-tailed Student’s *t*-tests were performed. *p* < 0.05 was considered statistically significant. The data were analyzed and graphed using GraphPad Prism 6.

## Results

### Method Validation

**FIGURE 1:**
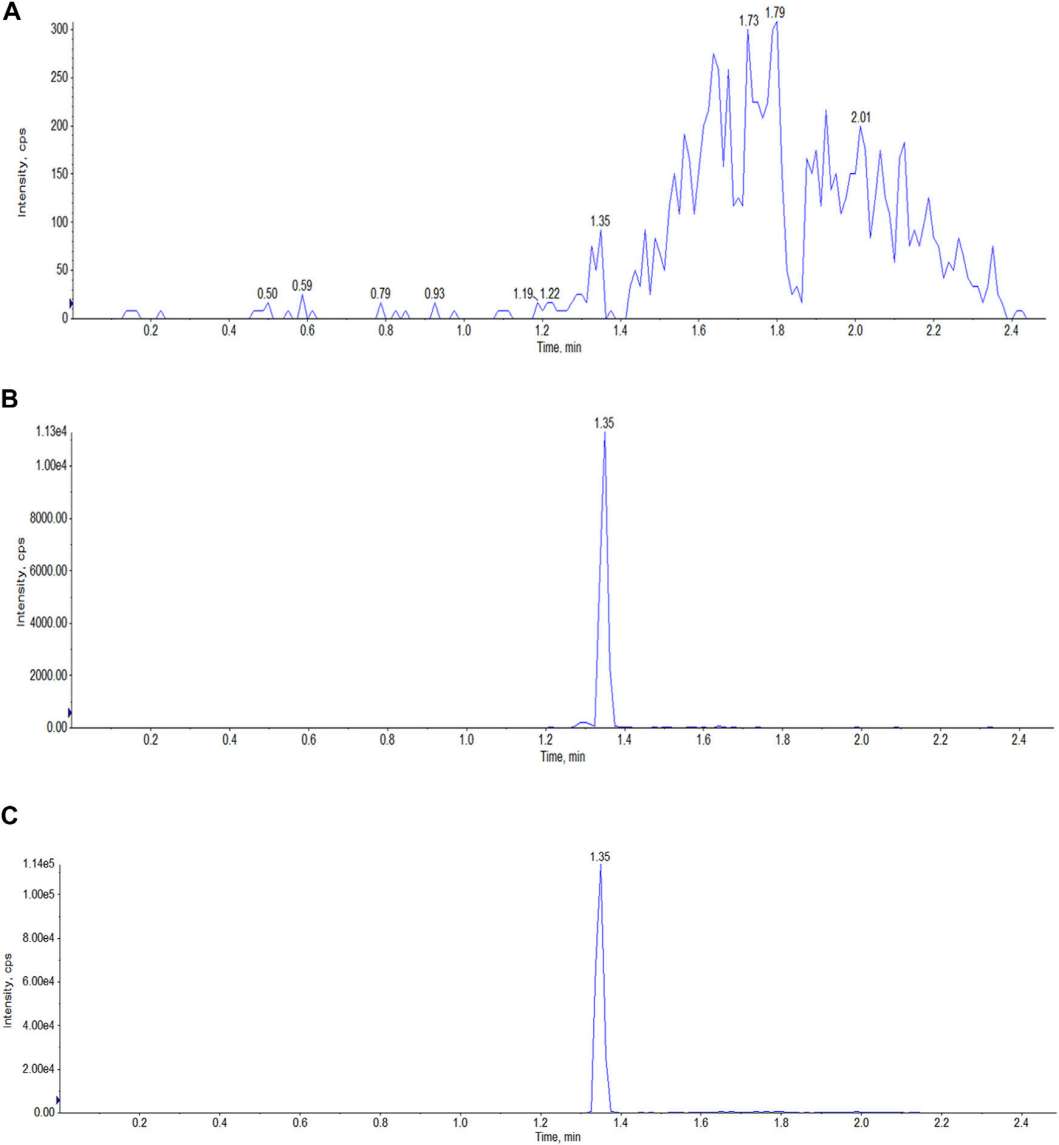
Typical LC-MS/MS MRM chromatograms of Indo5 (*m/z* 431.165/232.2) and IS (*m/z* 472.3/436.3) in plasma. **(A)** blank plasma; **(B)** Indo5; **(C)** IS.

*Selectivity*. Six blank samples of each matrix (plasma and tissues) were analyzed to evaluate the selectivity of our method. The samples were prepared in accordance with the method we developed. The blank samples were spiked with a standard solution of Indo5 and IS, and then analyzed; typical MRM chromatograms are shown in Figure 1. Our method demonstrated high selectivity given that there was no interference in the analyte peaks with those obtained using standards or the IS.

*Linearity*. Linearity was tested using calibration curves by spiking blank plasma and tissues at eight concentration levels. All of the calibration curves for Indo5 in plasma or tissue were linear over the concentration range 1–500 ng/ml with a correlation coefficient (*r*) > 0.99 (Table 1).

**TABLE 1:** Calibration curve and correlation coefficients of Indo5 in biological samples.

Matrix	Calibration curve	Correlation coefficient (R)
Plasma	y = 1.0016x + 0.1253	0.9992
Liver	y = 0.00101055x + 0.000254539	0.9976
Kidney	y = 0.000976907x + 0.00151646	0.9957
Heart	y = 0.00102639x + 0.000248108	0.9968
Lung	y = 0.000973661x − 0.000100517	0.9938
Stomach	y = 0.001531x + 0.00403645	0.9947
Small intestine	y = 0.00284016x + 0.00909608	0.9968
Large intestine	y = 0.00233707x + 0.00832539	0.9916
Spleen	y = 0.00258944x + 0.0060807	0.9964
Brain	y = 0.00421543x + 0.0034438	0.995
Testis	y = 0.00713123x + 0.0174964	0.993
Small-intestine content	y = 0.00258989x + 0.00451089	0.9976
Large-intestine content	y = 0.00384958x + 0.00737813	0.9951

*Accuracy and precision*. Accuracy and precision were evaluated at LLOQ, as well as low, medium, and high levels, with five replicates, on three separate days. The accuracy of detecting Indo5 in plasma and tissues was 85.5–112.5%. Within-run and between-run precision was <15% (Table 2). These results all met the criteria set by the FDA.

**TABLE 2:** Accuracy, within-run and between-run precision for Indo5 in rat plasma and tissues.

Matrix	QC level	Nominal concentration (ng/ml)	Accuracy (%)	Precision (CV^a^ %)	
				Within-run (*n* = 5)	Between-run (*n* = 5)
Plasma	LLOQ^b^	1	88.3	4.3	14
	LOW	5	92	7.9	11.7
	MEDIUM	100	90	6.6	8.4
	HIGH	500	97	2	5.2
Liver	LLOQ	1	86.1	12.4	13.2
	LOW	5	105.8	1.2	9.1
	MEDIUM	100	94.4	2	4.8
	HIGH	500	101.2	3.4	6
Kidney	LLOQ	1	110	5.4	14.6
	LOW	5	103	6.8	3.6
	MEDIUM	100	98.4	7.4	13.9
	HIGH	500	102.7	2.6	3.9
Heart	LLOQ	1	86.5	12.7	12.9
	LOW	5	106.2	9.4	11.7
	MEDIUM	100	111.3	4.7	6.4
	HIGH	500	97.9	2.7	4.1
Lung	LLOQ	1	85.8	12.7	12.8
	LOW	5	100.5	7.8	10.9
	MEDIUM	100	93.2	10.4	11.6
	HIGH	500	97.2	6.3	9.5
Stomach	LLOQ	1	85.5	14.3	13.9
	LOW	5	107.7	6.1	14.1
	MEDIUM	100	107.4	6	7.5
	HIGH	500	101.7	8.6	3.2
Small intestine	LLOQ	1	112.4	9.7	14.7
	LOW	5	94.6	2.6	6.5
	MEDIUM	100	104.2	3.1	3.7
	HIGH	500	102.9	2.5	3.9
Large intestine	LLOQ	1	113.5	4.1	9.3
	LOW	5	91.4	2.7	8.9
	MEDIUM	100	98.3	4.6	10.9
	HIGH	500	106.6	3.8	6.5
Spleen	LLOQ	1	87.6	10.3	9.6
	LOW	5	91.3	3.5	3.9
	MEDIUM	100	94.2	2.7	10.1
	HIGH	500	105.9	3.3	7.5
Brain	LLOQ	1	88.6	12.8	12.5
	LOW	5	112.1	8	11.5
	MEDIUM	100	95.4	3.3	10.7
	HIGH	500	101	4.3	5.1
Testis	LLOQ	1	111.8	8.8	13.9
	LOW	5	99	3.6	9.8
	MEDIUM	100	104.3	8.5	10.3
	HIGH	500	102.9	3.2	2.4
Small-intestine content	LLOQ	1	111	14.7	12.2
	LOW	5	92.7	7.2	10.4
	MEDIUM	100	106.4	2.4	2.9
	HIGH	500	98.3	4	3.7
Large-intestine content	LLOQ	1	89.2	13.2	13.9
	LOW	5	93.6	8.6	10.9
	MEDIUM	100	106.2	3.3	9
	HIGH	500	102.2	4.7	6.2

a CV:coefficient of variation.

b LLOQ:lower limit of quantification.

*Recovery and matrix-effect*. Recovery was calculated by comparing the analytical results for the extracted samples with the corresponding extracts of blanks spiked with Indo5 after extraction. The recovery effect of Indo5 at three QC levels ranged from 87.3 to 95.7% in plasma, and from 85.6 to 105.2% in the tested tissue homogenates (Table 3). The extraction recovery of IS in plasma and tissues was between 95.9 and 103.4%, indicating that protein precipitation as the sample preparation resulted in high and reproducible extraction efficiencies. The matrix effect of Indo5 at three QC levels ranged from 91.2 to 98.4% in plasma, and from 90.7 to 106.1% in the tissue homogenates, and the matrix effect of the IS in plasma and the tissues was between 93.6 and 104.5%, which indicated that there was no significant ion suppression or enhancement in the LC-MS/MS method (data not shown).

**TABLE 3:** Recovery of Indo5 in rat plasma and tissues.

Matrix	QC level	Recovery % (CV^a^; *n* = 5)
Plasma	LOW	87.3 (3.8%)
	MEDIUM	95.7 (5.9%)
	HIGH	94.1 (4.0%)
Liver	LOW	92.7 (5.8%)
	MEDIUM	96.5 (2.8%)
	HIGH	103.2 (5.4%)
Kidney	LOW	94 (4.7%)
	MEDIUM	101 (3.3%)
	HIGH	98.4 (6.1%)
Heart	LOW	88.9 (2.4%)
	MEDIUM	102.9 (2.0%)
	HIGH	100.9 (5.7%)
Lung	LOW	85.6 (6.5%)
	MEDIUM	89.3 (5.3%)
	HIGH	94.3 (2.6%)
Stomach	LOW	95.4 (3.5%)
	MEDIUM	104 (6.0%)
	HIGH	93.1 (7.0%)
Small intestine	LOW	86.2 (4.8%)
	MEDIUM	89 (7.3%)
	HIGH	94.7 (3.1%)
Large intestine	LOW	89.5 (1.5%)
	MEDIUM	95.2 (3.0%)
	HIGH	101.2 (5.6%)
Spleen	LOW	88.8 (4.4%)
	MEDIUM	96.3 (3.7%)
	HIGH	105.2 (2.3%)
Brain	LOW	91.6 (3.2%)
	MEDIUM	96.9 (4.6%)
	HIGH	104.3 (5.4%)
Testis	LOW	93.9 (3.6%)
	MEDIUM	96 (2.2%)
	HIGH	102.3 (1.4%)
Small-intestine content	LOW	86.4 (6.2%)
	MEDIUM	98.1 (3.1%)
	HIGH	99.4 (5.5%)
Large-intestine content	LOW	86 (7.9%)
	MEDIUM	90.7 (5.2%)
	HIGH	100.7 (3.4%)

a CV:coefficient of variation.

### Plasma Distribution

**FIGURE 2:**
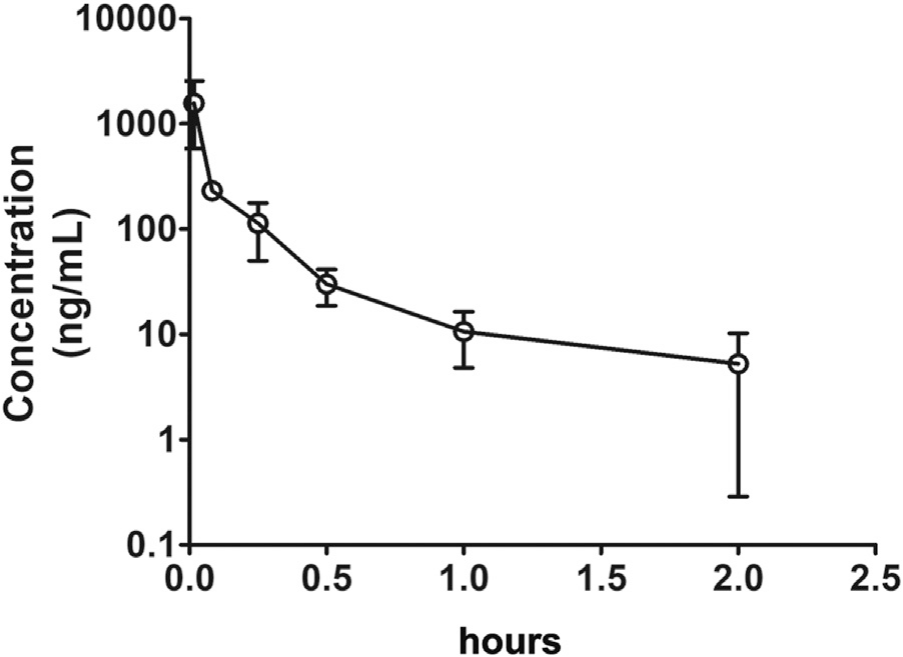
Plasma concentration–time profiles after intravenous administration of Indo5 (1 mg/kg) in rats (*n* = 3). Data are presented as the mean ± SD.

**FIGURE 3:**
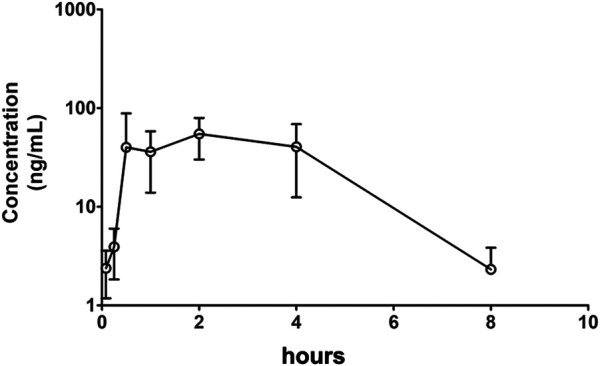
Plasma concentration–time profiles after oral administration of Indo5 (100 mg/kg) in rats (*n* = 3). Data are presented as the mean ± SD.

After IV (1 mg/kg in DMSO) or oral (100 mg/kg in 0.5% CMC-Na) administration of Indo5 to rats in each treatment group (*n* = 3), the plasma concentrations of Indo5 were determined using LC-MS/MS. The data obtained from each group were averaged. Figures 2, 3 show the mean plasma concentration–time curves for Indo5 after IV and oral administration, respectively. The corresponding pharmacokinetic parameters, calculated using a non-compartmental model are summarized in Table 4.

**TABLE 4:** Main pharmacokinetic parameters of Indo5 following intravenous or oral administration.

Parameters	Intravenous administration (IV) 1 mg/kg	Oral administration (p.o.) 100 mg/kg
t_1/2_ (h)	0.63 ± 0.11	1.25 ± 0.24
T_max_ (h)	0.0167	2.0 ± 0.48
C_max_ (ng/ml)	1,565.3 ± 286.2	54.7 ± 10.4
AUC_0-t_ (h*ng/ml)	158.6 ± 27.9	251.5 ± 43.5
AUC_0-∞_ (h*ng/ml)	163.4 ± 28.3	255.7 ± 44.9
Vd (ml/kg)	5,588 ± 9,433.8	706349 ± 106941.2
CL (ml/h/kg)	6,121.6 ± 1,124.8	391056.4 ± 77,945.8
MRT_0-t_ (h)	0.18 ± 0.04	2.87 ± 0.61
MRT_0-∞_ (h)	0.26 ± 0.06	2.99 ± 0.63

After IV administration, the plasma concentrations of Indo5 were well above the LLOQ within 2 h but, at 4 h, Indo5 was not detected. For IV injection, T_max_ was 1 min, C_max_ was 1,565.3 ± 286.2 ng/ml, AUC_0–t_ was 158.6 ± 27.9 h⋅ng/ml, AUC_0–∞_ was 163.4 ± 28.3 h⋅ng/ml, MRT_0–t_ was 0.18 ± 0.04 h, and MRT_0–∞_ was 0.26 ± 0.06 h. T_1/2_ was 0.63 ± 0.11 h, indicating that Indo5 had a short half-life *in vivo*. Moreover, Indo5 displayed a high systemic CL (6,121.6 ± 1,124.8 ml/h/kg) at the tested dose.

After oral administration, the plasma concentrations of Indo5 were above the LLOQ within 8 h. For oral administration, T_max_ was 2.0 ± 0.48 h, C_max_ was 54.7 ± 10.4 ng/ml, t_1/2_ was 1.25 ± 0.24 h, AUC_0–t_ was 251.5 ± 43.5 h⋅ng/ml, AUC_0–∞_ was 255.7 ± 44.9 h⋅ng/ml, MRT_0–t_ was 2.87 ± 0.61 h, MRT_0–∞_ was 2.99 ± 0.63 h, and CL was 391056.4 ± 77,945.8 ml/h/kg. According to the value for AUC_0–∞_ for oral administration and IV injection at 100 mg/kg, the bioavailability of Indo5 was 1.59%.

These results suggested that Indo5 was rapidly cleared from the plasma and had low bioavailability.

### Tissue Distribution

**FIGURE 4:**
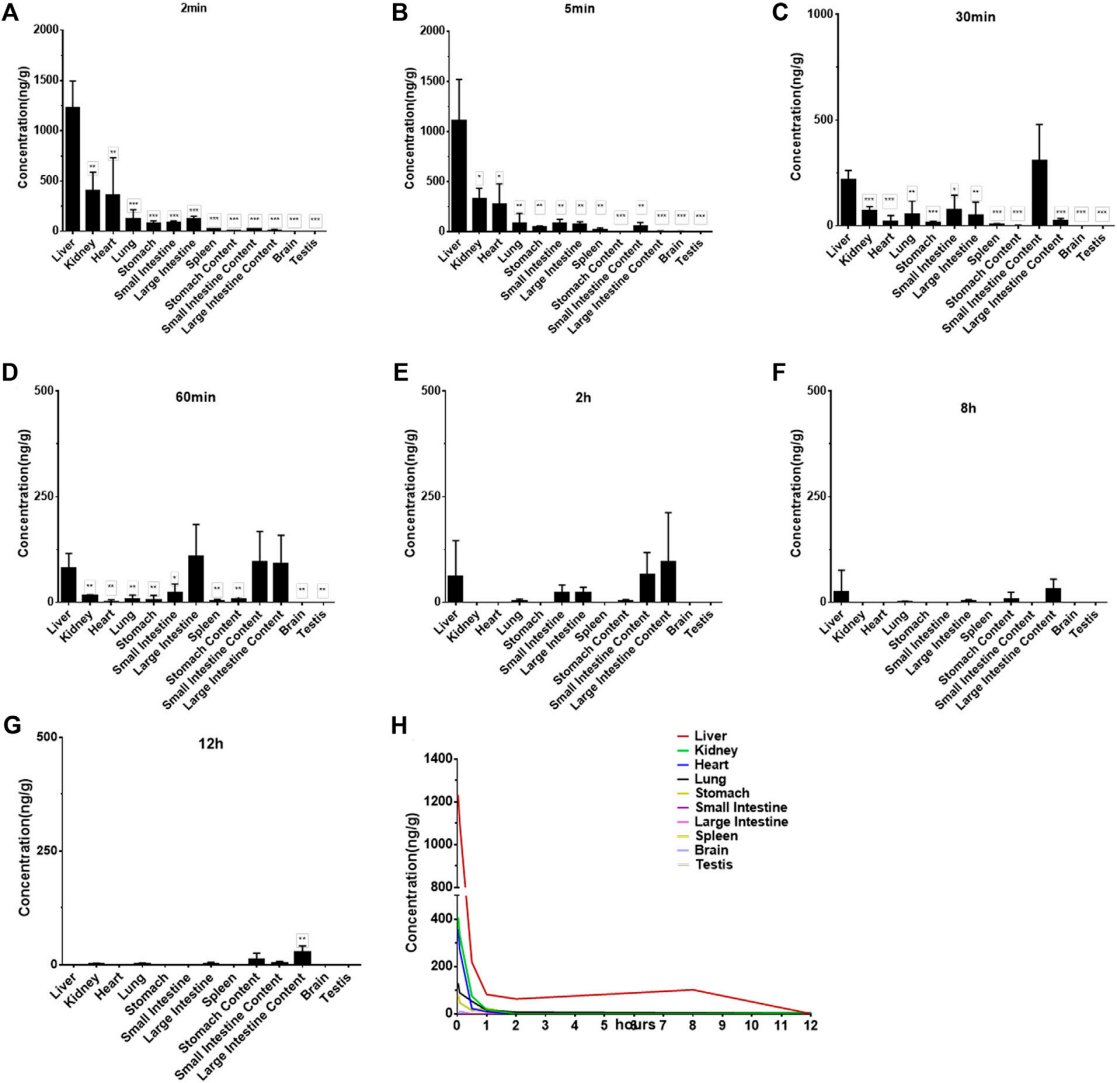
Indo5 concentration in different tissues following intravenous administration (1 mg/kg) (*n* = 4) at the indicated time points in rats **(A–G)**. Data are presented as the mean ± SD. Two-tailed t tests were performed. *p < 0.05, **p < 0.01, ***p < 0.001 when compared to Indo5 concentration in the liver. **(H)** concentration–time profiles of Indo5 in the indicated tissues.

The concentrations of Indo5 after IV administration (1 mg/kg) in the liver, kidneys, lungs, heart, spleen, stomach, large intestine, small intestine, brain, and testes at the indicated times in rats are shown in Figure 4. Indo5 was distributed rapidly and widely in the tested tissues in the following order: liver > kidneys ≈ heart > lungs ≈ large intestine ≈ small intestine ≈ stomach > spleen > brain ≈ testes. Indo5 was eliminated quickly from the tested tissues, and at 8 h, it was not detectable anymore in the kidneys, heart, lungs, large intestine, small intestine, stomach, spleen, brain, or testes. Notably, the hepatic Indo5 concentration was higher than that in other tissues, and it was still detectable in the liver 8 h post administration.

**FIGURE 5:**
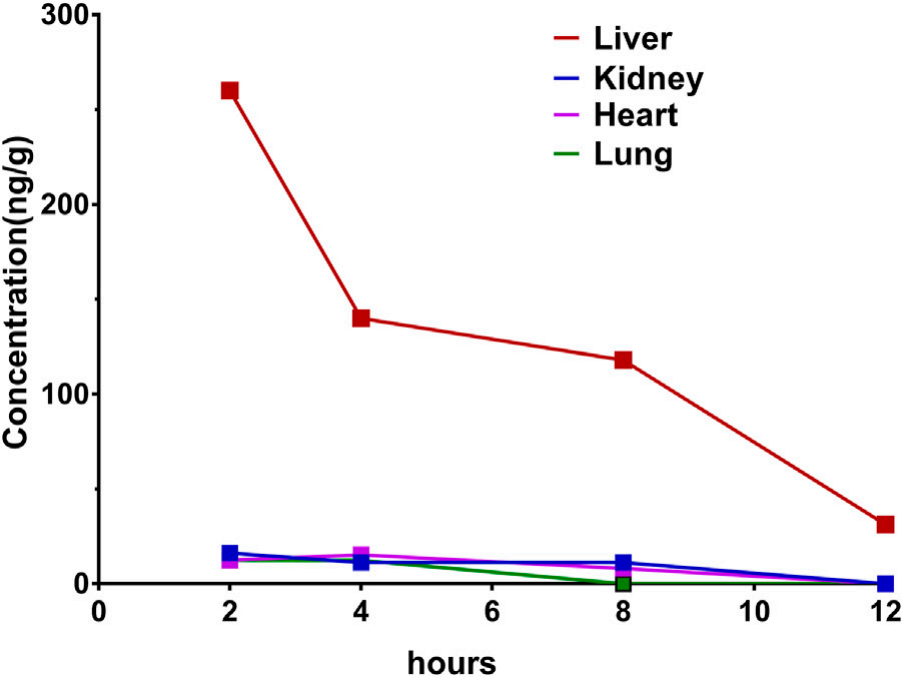
Concentration–time profiles after oral administration of Indo5 in the rat liver, kidneys, heart, and lungs at a dose of 100 mg/kg (*n* = 3).

To verify the liver enrichment of Indo5, we measured the Indo5 concentration in the tissues after oral administration. Consistent with the results following IV administration, the highest concentration of Indo5 was found in the liver, and it was detectable 8 h post administration (Figure 5).

Taken together, these results suggested that Indo5 displayed an accumulated distribution in the liver *in vivo*.

### Toxicity

**FIGURE 6:**
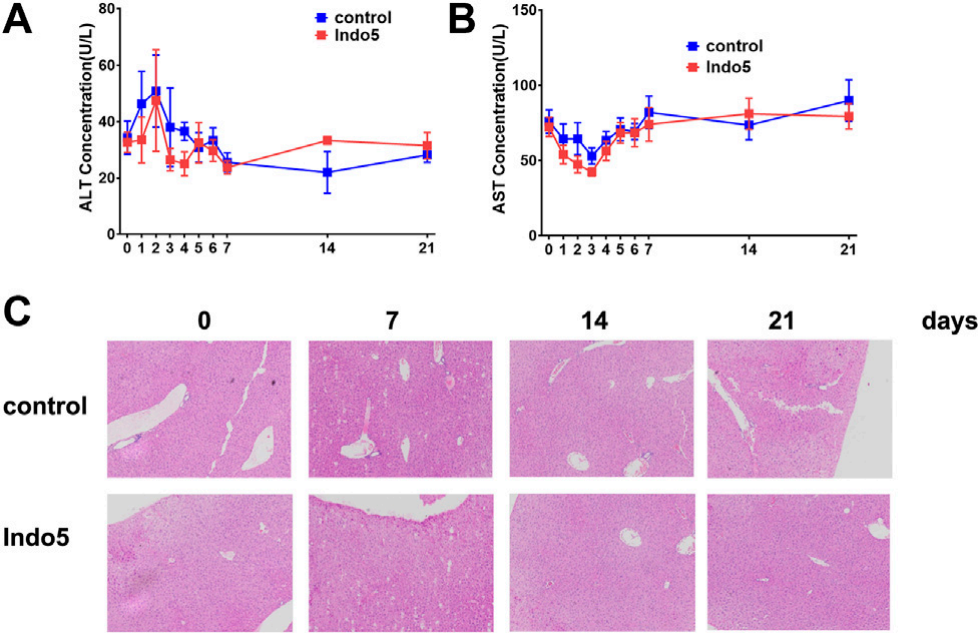
Effects of continuous administration of Indo5 for 21 days on liver injury in mice. The mice were orally administered with Indo5 (60 mg/kg, once daily) for 21 days (*n* = 6). Serum levels of ALT **(A)** and AST **(B)** were measured at the indicated time points. At the indicated time, the mice were killed for histopathological examination of liver tissue **(C)**. Data are presented as the mean ± SD. Two-tailed t tests were performed. *p < 0.05 were regarded as statistically significant, compared with control group.

**FIGURE 7:**
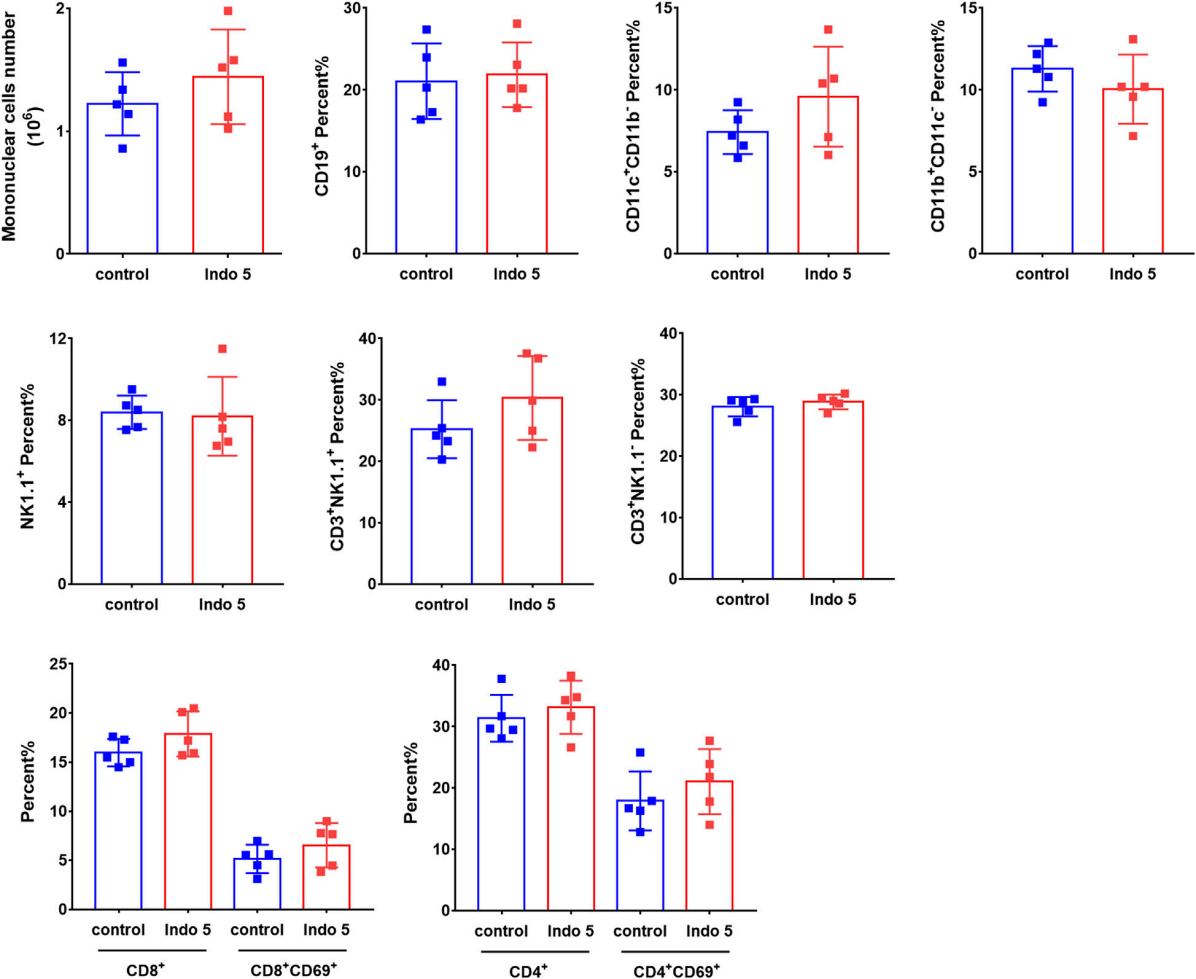
Effects of continuous administration of Indo5 for 21 days on the number and constitution of immune cells in the liver. The mice were orally administered with Indo5 (60 mg/kg, once daily) for 21 days (*n* = 6). On day 21, they were killed, and hepatic mononuclear cells were isolated. The number of liver mononuclear cells and frequencies of T cells (CD3^+^NK1.1^−^), NK cells (CD3^−^NK1.1^+^), NKT cells (CD3^+^NK1.1^+^), B cells (CD19^+^), DCs (CD11c^+^CD11b^−^), myeloid cells (CD11b^+^CD11c^−^), T-cell subsets (CD4^+^, CD8^+^), and activated T cells (CD4^+^CD69^+^, CD8^+^CD69^+^) were measured using flow cytometry. Data are presented as the mean ± SD. Two-tailed t tests were performed. All *p* values were greater than 0.05. There was no statistical significance in the number or frequencies of immune cells in the liver between the administration group and the control group.

Given that Indo5 was most distributed in the liver and had significant antitumor activity in a xenograft model and hepatic orthotopic model in mice (Luo et al., 2019), we investigated if continuous administration of Indo5 leads to liver injury. Indo5 was orally administered (p.o.) once daily for 21 days, and we evaluated its toxicity. No obvious weight loss or other symptoms were observed with continuous injection of Indo5. The overall health of the animals was not adversely affected (data not shown). The levels of alanine aminotransferase (ALT) and aspartate aminotransferase (AST) in serum did not change significantly after Indo5 administration (Figures 6A,B; *p* > 0.05). Histological analysis of liver tissue revealed no pathological changes in Indo5-treated mice compared with the solvent-control group, *i.e.*, a normal lobular architecture with a central vein and radiating hepatic cords were observed (Figure 6C). Often, liver injury leads to infiltration or activation of immune cells in liver tissue. We thus investigated the number of liver mononuclear cells and frequencies of various immune cells, including T cells, natural killer cells, natural killer T cells, B cells, dendritic cells, and myeloid cells. The frequencies of T-cell subsets (CD4^+^, CD8^+^) and activated T cells (CD4^+^CD69^+^, CD8^+^CD69^+^) were also measured. Continuous administration of Indo5 for 21 days did not affect the number or frequencies of immune cells in the liver (Figure 7; *p* > 0.05).

These results suggested that Indo5 did not show obvious toxicity.

## Discussion

Here, we developed a series of validated HPLC-MS/MS methods to measure the Indo5 concentration in plasma and tissue homogenates. To investigate Indo5 absorption, we measured the concentrations of Indo5 in rat plasma at different time intervals. After IV injection of Indo5, T_max,_ and C_max_ were 1 min and 1,565.3 ± 286.2 ng/ml, respectively. C_max_ of Indo5 in the oral-administration group was 54.7 ± 10.4 ng/ml, and it occurred at 2.0 ± 0.48 h. The absolute oral bioavailability of Indo5 in rats was 1.59%. The tissue-distribution study suggested that Indo5 accumulated in the liver and could not cross the blood–brain or blood–testes barriers. The toxicity study suggested that continuous injection of Indo5 for 21 days did not lead to liver injury because the levels of ALT and AST were unchanged, normal histological architecture of the liver was preserved, and normal number and frequency of immune cells were found in the liver. Our findings provide a more complete understanding of the biological actions of Indo5, and also provide insights for the future modification of Indo5 formulations to increase bioavailability.

In general, the oral route for drug administration is considered more attractive than the IV route because of its greater convenience for patients and pharmacoeconomic advantages (Liu et al., 1997; Schellens et al., 2000; Oostendorp et al., 2011)*.* We observed a low oral bioavailability of Indo5 (1.59%) at 100 mg/kg, which might have been due to the low solubility and stability of this compound. Previous studies have reported that compounds with low solubility show low oral bioavailability, for example, paclitaxel (Rowinsky et al., 1994; Huizing et al., 1995), wogonin (Talbi et al., 2014), and silymarin (Bijak, 2017; Abenavoli et al., 2018). Low bioavailability is a major obstacle for biomedical applications of drugs. Therefore, finding a new formulation with high solubility in water and low cost is necessary. In recent years, considerable efforts have been made to improve the oral absorption of insoluble drugs. Drug modifications, including the formation of salts, esters, and complexes with hydrophilic excipients, have been used to optimize silymarin solubility (Chen et al., 2011; Javed et al., 2011; Bonifácio et al., 2014). Most formulation strategies refer to complexation with cyclodextrins or phospholipids (“phytosomes”), solid dispersions stabilized by biocompatible polymers, microemulsions, nanoemulsions, lipid-based delivery systems, biodegradable polymeric nanoparticles, and inorganic nanomaterials (Bilensoy et al., 2008; Dahmani et al., 2012; Li et al., 2015b; Hussain et al., 2016; Yao et al., 2017; Ma and Williams, 2018). Further investigations are needed to improve the oral bioavailability of Indo5.

Investigating the tissue-specific distribution of a compound can reveal much information, including its potential side effects or possibilities for drug repositioning (Esch et al., 2015). Here, we described the biodistribution pattern of Indo5. The highest concentration of Indo5 was detected in the liver after intravenous and oral dosing. Furthermore, Indo5 was not able to cross the blood–brain barrier or blood–testes barriers. The analysis of the toxicity of Indo5 in the liver suggested that continuous oral administration of Indo5 for 21 days had no effect on serum levels of ALT or AST. Moreover, normal tissue architecture was preserved in the Indo5-treated group, and the number of hepatic mononuclear cells, the frequency of different immune-cell subsets, and the activation status of T cells in the liver were unchanged. These data suggest that, even though Indo5 is most distributed in the liver, it does not cause significant toxicity. Taken together with the result that Indo5 demonstrates antitumor activity and improves overall survival in a mouse hepatic orthotopic model (Luo et al., 2019), our findings imply that Indo5 may be helpful in HCC treatment.

## Conclusion

Indo5, a selective inhibitor of c-Met and Trks, is mostly distributed in the liver *in vivo*. It shows no obvious toxicity or side effects in the liver, which further supports its development as an antitumor treatment for HCC patients.

## Data Availability

The raw data supporting the conclusions of this article will be made available by the authors, without undue reservation.
